# The Murine *Reg3a* Stimulated by *Lactobacillus casei* Promotes Intestinal Cell Proliferation and Inhibits the Multiplication of Porcine Diarrhea Causative Agent *in vitro*

**DOI:** 10.3389/fmicb.2021.675263

**Published:** 2021-06-18

**Authors:** Yongfei Bai, Yanmei Huang, Ying Li, Bingbing Zhang, Cuihong Xiao, Xilin Hou, Liyun Yu

**Affiliations:** ^1^College of Life Science and Technology, Heilongjiang Bayi Agricultural University, Daqing, China; ^2^Colleges of Animal Science and Technology, Heilongjiang Bayi Agricultural University, Daqing, China

**Keywords:** *Lactobacillus casei*, antimicrobial peptides, murine *Reg3a*, intestinal cells proliferation, porcine diarrhea causative agent

## Abstract

*Lactobacillus casei* (*L. casei*), a normal resident of the gastrointestinal tract of mammals, has been extensively studied over the past few decades for its probiotic properties in clinical and animal models. Some studies have shown that some bacterium of *Lactobacillus* stimulate the production of antimicrobial peptides in intestinal cells to clear enteric pathogens, however, which antimicrobial peptides are produced by *L. casei* stimulation and its function are still not completely understood. In this study, we investigated the changes of antimicrobial peptides’ expression after intragastric administration of *L. casei* to mice. The bioinformatics analysis revealed there were nine genes strongly associated with up-regulated DEGs. But, of these, only the antimicrobial peptide m*Reg3a* gene was continuously up-regulated, which was also confirmed by qRT-PCR. We found out the m*Reg3a* expressed in engineering *E.coli* promoted cell proliferation and wound healing proved by CCK-8 assay and wound healing assay. Moreover, the tight junction proteins ZO-1 and E-cadherin in m*Reg3a* treatment group were significantly higher than that in the control group under the final concentration of 0.2 mg/ml both in Porcine intestinal epithelial cells (IPEC-J2) and Mouse intestinal epithelial cells (IEC-6) (*p* < 0.05). Surprisingly, the recombinant m*Reg3a* not only inhibited Enterotoxigenic *Escherichia coli* (ETEC), but also reduced the copy number of the piglet diarrheal viruses, porcine epidemic diarrhea virus (PEDV), porcine transmissible gastroenteritis virus (TGEV), and porcine rotavirus (PoRV), indicating the antimicrobial peptides m*Reg3a* may be feed additives to resist the potential of the intestinal bacterial and viral diarrhea disease.

## Introduction

The intestinal barrier is composed of the intestinal microbiota, mucus layer, intestinal epithelium, its intercellular junctions, and intestinal immune system. The intestinal microbiota is essential for the maturation and maintenance of the epithelial barrier by promoting epithelial cell proliferation, maintaining tight junctions, and mucus production (Abreu, 2010). *Lactobacillus casei* (*L. casei*), a normal resident of the gastrointestinal tract of mammals, has been extensively studied over the past few decades for its probiotic properties in clinical and animal models (McFarland et al., 2018; Riaz Rajoka et al., 2018). Many studies have shown that *L. casei* may have physical health benefits for humans and domestic animals. These probiotics are generally thought to stabilize the intestinal microbiota, inhibit the development of pathogenic microorganisms, and prevent or mitigate the course of diarrhea caused by pathogenic bacteria and viruses, as well as normalize the disturbance of intestinal motility (Toh et al., 2013; Hill et al., 2018). Also, several studies have shown that the *Lactobacillus* strain stimulates the production of antimicrobial peptides in intestinal cells to clear enteric pathogens (Liu et al., 2017; Wang J. et al., 2019). However, which antimicrobial peptides produced by *L. casei* stimulate the expression change of the antimicrobial peptides are still not completely understood.

The *Reg* gene family, C-type lectin, secreted by Paneth cell has powerful activity against bacteria and viruses in the gut. Depending on the primary structure, the *Reg* protein was classified into four types: I, II, III, and IV. Murine *Reg* family proteins can stimulate different types of cell and tissue proliferation and neogenesis, and also protect experimental animals from toxic injury and disease onset (Liu et al., 2008). The m*Reg3a* belongs to the superfamily of mice dependent lectin domain, and was originally cloned from a partially pancreatectomized rat pancreas cDNA library (Yonemura et al., 1984). The secreted *Reg3a* protein has been reported for early diagnosis of gastric and colorectal cancers as a biomarker (Nagaraj and Reverter, 2011), which play an important role for proliferation in liver cells and insulinoma cells. But it is also not understood whether the m*Reg3a* produced by *L. casei* stimulation promotes cell proliferation and repairs the injured intestinal cells or not.

In the present work, we mainly investigated the changes of antimicrobial peptide expression after orally administration to mice of *Lactobacillus casei*. The bioinformatics analysis uncovered what antimicrobial peptides were produced by *Lactobacillus casei* and the expression amount of the antimicrobial peptides. Then the higher expressed *Reg3a* was chosen to express *in vitro* to discuss its activity. We found out it promoted cell proliferative and wound healing proven by CCK-8 assay and wound healing assay. The expressed *Reg3a* not only inhibited ETEC, but also reduced the copy number of diarrheal viruses, PEDV, TGEV, and PoRV. We anticipate that this antimicrobial peptide protects animals from enteric pathogens that are transmitted through the mucosa and can repair damaged intestinal cells.

## Materials and Methods

### Bacterial Strains and Growth Conditions

*Lactobacillus casei* (ATCC-334) was purchased from China Center of Industrial Culture Collection (CICC) and grown anaerobically at 37°C without agitation in MRS broth medium (Difco).

### Animal and Sample Preparation

A total of 24 mice from 2 litters (Balb/c, SPF, 4-week-old) were obtained from the Vital River Laboratories (Beijing, China). All mice in this study were chosen from one delivery room and had similar genetic backgrounds and husbandry practices. These mice were allocated randomly to two groups (NTC: no feeding *L.casei*, TOA: feeding *L.casei* on days 1–30) for 35 days. The mice in two groups were placed in two pens with a similar environment. Twelve mice in the NTC group were fed the basic diet without any probiotics for 30 days. For the treatment group, twelve mice in the TOA group were treated with the protocol described by Li-Juan Wen (Wen et al., 2012). *L.casei* cells (5 × 10^11^ CFU/ml) in 200 ml PBS were orally administered daily on days 1–30 and treated according to the animal protocols approved by the Institutional Animal Care and Use Committee (IACUC). The use of animals for this experiment was approved by the Heilongjiang Bayi Agricultural University Institutional Animal Care and Use Committee.

For the Transcriptome sequencing, 3 mice’s small intestine tissue samples located in the stomach pyloric to ileocecal were collected in cryopreservation tube (Axygen) on days 10, 20, 30, and 35 in the TOA group and on days 10, 20, and 30 in the NTC group. After sample collection, the samples were quickly placed into the sterile tubes, then threw into liquid nitrogen for half an hour, and then stored at −80°C until sequencing.

### Transcriptome Sequencing for Small Intestine Tissue Sample

PolyA^+^ mRNA was extracted by using Magnosphere^TM^ UltraPure mRNA Purification Kit (No.9186, Takara, Shiga, Japan) following the manufacturer’s protocol. RNA quality was assessed on an Agilent 2100 Bioanalyzer (Agilent Technologies, Palo Alto, United States) and checked by using 1.2% RNase free agarose gel electrophoresis. The RNA concentration was measured by NanoDrop one instrument (Thermo Fisher Scientific, Waltham, United States). The enriched mRNAs were fragmented by using fragmentation buffer and reverse transcribed into cDNA with random primers. The second-strand cDNA was synthesized by DNA polymerase I, RNase H, dNTP (dUTP instead of dTTP), and buffer. Next, the cDNA fragments were purified with QiaQuick PCR extraction kit (Qiagen, Venlo, Netherlands), the end group of which was modified by poly (A) and ligated to Illumina sequencing adapters. Then UNG (Uracil-N-Glycosylase) was used to digest the second-strand cDNA. The digested products selected in size by agarose gel electrophoresis were amplified via PCR and sequenced by using Illumina HiSeqTM 2500 (Shanghai Personal Biotechnology Co., Ltd., Shanghai, China). All the raw sequencing data were submitted to the NCBI Sequence Read Archive (SRA) database under accession No. PRJNA719601.

### Data Processing

In order to get high quality clean reads, the sequencing reads were further filtered by fastp (version 0.18.0) (Chen et al., 2018). The Alignment with reference genome was conducted by “-rna-strandness RF” command in HISAT2 (version 2.1.0) (Kim et al., 2015) and other parameters set as a default. The reconstruction of transcripts was carried out by software Stringtie (version 1.3.4) (Pertea et al., 2015, 2016), which, together with HISAT2, allowed biologists to identify new genes and new splice variants of known genes. Transcripts abundances were quantified by software StringTie in a reference-based approach.

To identify the differentially expressed transcripts across experiment groups, the DESeq2 package was used (Love et al., 2014). We identified the mRNAs with fold change > 2 and *p* < 0.05 in a comparison as significant DEGs, and the results were visualized by R software (version 3.6.1) with ggplot2 (version 3.3.3) and pheatmap packages (version 1.0.12). To assess functional enrichment, Gene Ontology (GO)’s Biological Processes term and Kyoto Encyclopedia of Genes and Genomes (KEGG), as well as the pathway analyses of DEGs, were performed by using the ClusterProfiler package in Bioconductor^1^ (Yu et al., 2012). The results were visualized by R software with ggplot2 package (version 3.3.3).

In order to understand the expression of antimicrobial peptides at each time point, we analyzed the time trend of antimicrobial peptide expression. We followed the method described by Jason Ernst (Ernst and Bar-Joseph, 2006). In brief, it is to use the first time point as a control to calculate the multiple of the expression level of all samples relative to the first time point. Then we took the log_2_ value (multiple value of log_2_ treatment) for the expression multiple and visualized by R software with ggplot2 package.

### qRT-PCR

The total RNA 50 ng was used to carry out reverse transcription reactions by using HiScript III 1st Strand cDNA Synthesis Kit (Vazyme, Nanjin, China), then the cDNA in 20 μl ChamQ SYBR Color qPCR Master Mix (Low ROX Premixed, Vazyme, Nanjin, China) was amplified by qRT-PCR (LightCycler 96 real-time PCR system, Roche, Basel, Switzerland) with the designed primers (Table 1). The samples were run for 40 cycles (94°C for 30 sec, 58°C for 30 sec, and 72°C for 30 sec). The amplification specificity was confirmed by the analysis of melting curves. The relative gene expression values were calculated using the -2^ΔΔ*Ct*^ method of the LightCycler 96 Managing software (Roche, Basel, Switzerland).

**TABLE 1:** Primers for -2^ΔΔ*Ct*^ method qRT-PCR in this study.

**Gene name**	**Forward primer**	**Reverse primer**
m*Defa39*	CTTGTCCTCCTCTCTGCCCT	TGGTCCTCTTCCTCTGGCTG
m*Defa35*	CTCTCTGCCCTTGTCCTGCT	ATCATGAAGAGCAGACCCTTCT
m*Defa38*	CTTGTCCTCCTCTCTGCCCT	TCCTCTTCCTCTGGCTGCTC
m*Reg3a*	GCTGCCCCATGGGTTACAAG	TCCTGAGGGTCTCTTCTGGC
m*ZO1*	GAGATGTTTATGCGGACGG TGG	GTTTCCTCCATTGCTGTGCTCTTAG
m*E-cadh*	CTTTGAGGGATTCGTTGCAG AAGG	ATGTTTTTGTGAGGCAGCTCAGGAT
m*Ppia*	CCACTGTCGCTTTTCGCCGC	TGCAAACAGCTCGAAGGAGACGC
s*ZO1*	CCCCAGGGACTGGAAGAAA TCAC	CCCCTCCACTTCCTCTAGCCAAG
s*E-cadh*	TGAGAAATGAGTGTGCTTTT GTGCC	TGGCCAGACTAAGACATGAACCTC
s*Ppia*	ACACAAACGGTTCCCAGTTT TTCAT	TCCATGGCTTCCACAATATTCATGC

### Western Blot Analysis of *Reg3a* in Small Intestine Tissue Sample

Western blot analysis detected the protein m*Reg3a* in small intestine tissue sample. The homogenate of small intestine tissues stimulated by *L.casei* were treated by 0.4 ml lysis buffer (Solarbio, Peking, China) with 1 mM phenylmethanesulfonyl fluoride (PMSF). The supernatants were collected by centrifugation at 10000 × g for 5 min at 4°C. The protein samples (20 μg) were electrophoretically separated in 10% SDS-PAGE gels. Then, the protein samples were transferred to Nitrocellulose (NC) membranes. After blocking for 1 h at room temperature, the membranes were incubated overnight at 4°C with the following primary antibodies: anti-*Reg3a* antibody (Affinity, Cincinnati, OH, United States) and anti-beta Actin antibody. The NC membranes were washed and incubated with a secondary antibody (Abcam, Cambridge, England) for 1 h at room temperature. Finally, chemiluminescence detection was performed with Immobilon ECL Ultra Western HRP Substrate (Millipore, Massachusetts, United States) on a MicroChemi imaging system (DNR, Israel). The intensity of each band was quantified in ImageJ software. Each band was standardized by internal reference gene (Beta Actin), and then compared with the control group.

### Prokaryotic Expression and Purification of Antimicrobial Peptides

The DNA fragments encoding the antimicrobial peptides were amplified by RT-PCR with the specific primers. The PCR product was inserted into the vector pET-32a to construct the recombinant plasmid after digestion by *Bam*HI and *Hin*dIII. PCR amplification was performed as follows: 94°C for 2 min; 30 cycles of 94°C for 30 s, 58°C for 30 s, and 72°C for 45 s; and 72°C for 5 min of final extension. After heat shock transformation, the recombinant plasmid was transformed into *E.coli* strain BL21 and selected on LB medium plates containing 100 μg/ml of Ampicillin (Amp) and incubated aerobically at 37°C for 24 h.

In order to obtain the antimicrobial peptides expressed by prokaryotic cells, the recombinant *E.coli* was cultured in LB medium containing ampicillin 100 μg/ml incubated in anaerobic condition at 37°C for 24 h. IPTG was added in medium when the OD600 was 0.4. After centrifugation at 6000 × g, the bacteria were washed three times by sterile PBS solution. Subsequently, the antimicrobial peptides expressed by recombinant *E.coli* were purified by using the Ni-NTA Agarose (QIAGEN, Dusseldorf, Germany) according to the manufacturer’s instructions. SDS-PAGE and Western Blot (Peroxidase-conjugated anti-6X His tag antibody, Abcam, Cambridge, England) were employed to determine the accuracy of expressed antimicrobial peptides.

### Co-culture of Antimicrobial Peptides With ETEC or Porcine Diarrhea Viruses

To understand its function on Enterotoxigenic *Escherichia coli* (ETEC) and viruses, the antimicrobial peptides (2, 0.5 mg/ml) were co-cultured with ETEC. The ETEC strain K88 (C83902), K99 (C83912), and 987P (C83916) (5 × 10^5^ CFU/ml) were plated separately into a 96-microtiter plate (Standard flat-bottom wells, Corning, Corning, United States) with 50 μl per well. Then the LB medium as the negative control or antimicrobial peptides diluted with the LB medium was added in each well to stimulate the cells. Then, the plate was incubated in anaerobic conditions at 37°C for 12 h. The OD 600 values were determined by an autoreader (Tecan, Männedorf, Switzerland) at 4, 6, 9, and 12 h, respectively.

To understand the inhibitory effect of antimicrobial peptides on viruses, the virus TGEV (strain AHHF, GeneBank: KX499468.1), PEDV (strain LNct2, GeneBank: KT323980), and PoRV (strain HLJ/15/1, GeneBank: KU886317.1) infect Vero cells with MOI = 1 in 6-wells cell culture plates (NEST, Wuxi, China). Antimicrobial peptides (2, 0.5 mg/ml) were added in plates when cells displayed a Cytopathic effect (CPE). After incubation for 24 h, the RNA was extracted from Vero cells by using TIANamp Virus RNA Kit (Tiangen, Peking, China). The absolute quantitative method qRT-PCR was employed to calculate the viral copy number with the designed primers (Table 2).

**TABLE 2:** Primers for absolute quantitative method qRT-PCR in this study.

**Name**	**Forward primer**	**Reverse primer**
PEDV	CAGAAATTTTGTCCTTCCTTC CGGC	GTCTAGTATGTAGAAGGCGACG GAACG
TGEV	GATCCATTCAGTTGTACAGAA GGACTAAG	CCCTGAAAGCAAAGTTAGAGTG ACA
PoRV	CGCGGAGCTAAACGTGAAAA	TTACTCTCCATAATTGCGTCTATG TTCTT

### CCK-8 Assay and Healing Assay

Porcine intestinal epithelial cells (Ipec-J2) [bought from Beijing Beina Chuanglian institute of Biotechnology (Peking, China)] and Mouse intestinal epithelial cells (IEC-6, ATCC CRL-1592) were cultivated in DMEM medium with 10% FBS (Fetal Bovine Serum, Gibco, CA, United States) and 1% PS (Penicillin-Streptomycin Solution, Gibco CA, United States). The cells grew to monolayer in 25 cm^2^ flasks (Corning, Corning, United States) at 37°C under the condition of 5% CO_2_ and 90% humidity. To assess cell proliferation, the Ipec-J2 cells or IEC-6 cells were seeded into 96-well culture plates (Standard flat-bottom wells, Corning, Corning, United States) in 100 μl with the final cell-number 7000 cells per well. The antimicrobial peptides with the final concentration of 0.05 and 0.2 mg/ml were added in plates. Then the plate was incubated at 37°C for 24 h in a humidified atmosphere containing 5% CO_2_. The plate continued to incubate for 2 h with 10 μl CCK-8 reagent (Biosharp, Peking, China) per well, then the plate was read at 450 nm using an autoreader (Tecan, Männedorf, Switzerland). For healing assay, the Ipec-J2 cells or IEC-6 cells was plated into the 6-well culture plates (Standard flat-bottom wells, Corning, Corning, United States) with 1 ml per well with cell-number in 5 × 10^6^ cells. Then the plate was incubated at 37°C in 5% CO_2_ for confluence rate to 80%. The cells were scratched by a pipette tip and washed with HBSS (Hyclone, Logan, Utah, United States) softly. And then antimicrobial peptides diluted with DMEM medium were added in each well with a final concentration of 0.05 and 0.2 mg/ml. Then the plate was incubated at 37°C for 24 h in 5% CO_2_. The wound area at the same magnification was measured under the microscope (CKX41, Olympus, Hataya, Japan).

### Western Blot Analysis of the Tight Junction Proteins

Western blot analysis detected the protein expression of ZO-1 and E-cadherin. The cells stimulated by antimicrobial peptides were treated by 0.25 ml lysis buffer (Solarbio, Peking, China) with 1 mM phenylmethanesulfonyl fluoride (PMSF). The supernatants were collected by centrifugation at 10000 × g for 5 min at 4°C. The protein samples (20 μg) were electrophoretically separated in 10% SDS-PAGE gels. Then, the protein samples were transferred to Nitrocellulose (NC) membranes. After blocking for 1 h at room temperature, the membranes were incubated overnight at 4°C with the following primary antibodies: anti-ZO 1 antibody (Affinity, Cincinnati, OH, United States), E-cadherin antibody (Affinity, Cincinnati, OH, United States), and anti-beta Actin antibody. The NC membranes were washed and incubated with a secondary antibody (Abcam, Cambridge, England) for 1 h at room temperature. Finally, chemiluminescence detection was performed with Immobilon ECL Ultra Western HRP Substrate (Millipore, Massachusetts, United States) on a MicroChemi imaging system (DNR, Israel). The intensity of each band was quantified in ImageJ software. Each band was standardized by internal reference gene (Beta Actin), and then compared with the control group.

### Statistical Analysis

Graphpad prism 8.0 software was used for statistical analysis of all the experimental data. The Student’s *t*-test was employed to evaluate differences between variations in comparison groups. Differences were considered significant when the *p* < 0.05.

## Results

### Identification of Differentially Expressed Genes (DEGs) in Comparison Groups

To evaluate the global picture of mice intestine transcriptomic response to *L.casei* feeding, almost fifty-million reads from each sample were obtained by Illumina HISeq 2500 platform with a Q20 value of 97.5 ± 0.3% and a Q30 value of 93.5 ± 0.2%. For further analysis, the high-quality clean reads were mapped to the reference Mus_musculus genome (GRCm38). In addition, the uniquely mapped ratio of 92.8% ± 0.3 was determined after quality control (Data not shown). The results suggested that these high-quality sequencing data were available for further expression level analysis.

**FIGURE 1:**
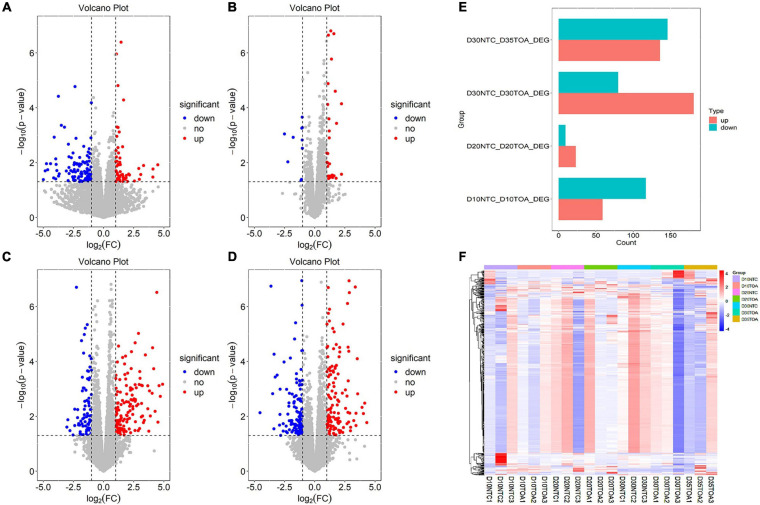
Differentially expressed analysis in comparison groups. The differential expression analysis in two groups was taken as follows: D10NTC vs. D10TOA group **(A)**, D20NTC vs. D20TOA group **(B)**, D30NTC vs. D30TOA group **(C)**, and D30NTC vs. D35TOA group **(D)** with *p* ≤ 0.05 and | FC| ≥ 2 and visualized by R software with ggplot2 package. Red dots mean up-regulated genes, blue dots mean down- regulated genes, and gray dots mean no difference genes. The DEGs details were visualized by bar-plot with ggplot2 package **(E)**, and the differential expression heat-map in every sample was visualized by R software with pheatmap package **(F)**. In bar-plot, red indicated the up-regulated gene, and green indicated the down-regulated gene in comparison groups. In heat-map, each column represented one sample, and each row referred to a gene. The color legend was on the top-right of the figure, and compound relative abundances were standardized prior to unsupervised hierarchical clustering of samples (rows). Red indicated the genes with higher expression relative to the geometrical means; blue indicated the genes with lower expression relative to the geometrical means. The color legend indicated the sample group.

To identify the differentially expressed transcripts across samples or groups, the Deseq2 package^2^ was used and the volcano plot was visualized by the ggplot2 package of R software. Briefly, the FC (Fold-change) values of each gene at different feeding times was compared with the control group; the threshold values *p* ≤ 0.05 and | FC| ≥ 2 were used to identify DEGs between the treating group and the control group at different time points (Figures 1A–D). The results showed that 176, 32, 261, and 282 genes changed in different comparison groups of D10NTC vs. D10TOA, D20NTC vs. D20TOA, D30NTC vs. D30TOA, and D30NTC vs. D35TOA. In detail, there were 59, 23, 181, 136 up-regulated genes and 117, 9, 80, 146 down-regulated genes identified in D10NTC vs. D10TOA, D20NTC vs. D20TOA, D30NTC vs. D30TOA, and D30NTC vs. D35TOA (Figures 1E,F).

### Gene Ontology (GO) Enrichment Analysis of DEGs in Comparison Groups

**FIGURE 2:**
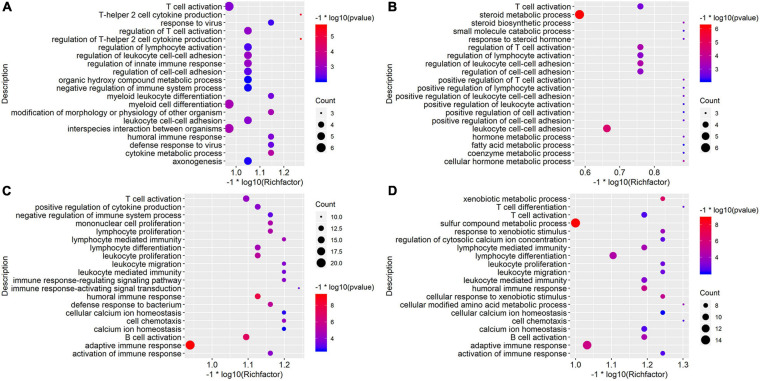
Gene Ontology (GO) enrichment analysis of up-regulated DEGs in comparison groups. The top 20 significant Biological Processes terms of up-regulated DEGs in D10NTC vs. D10TOA group **(A)**, top 20 significant Biological Processes terms of up-regulated DEGs in D20NTC vs. D20TOA group **(B)**, top 20 significant Biological Processes terms of up-regulated DEGs in D30NTC vs. D30TOA group **(C)**, and top 20 significant Biological Processes terms of up-regulated DEGs in D30NTC vs. D35TOA group **(D)**. Size and color of the bubbles are measured as count and *p-*value to represent the amount of DEGs enriched in these terms and the enrichment significance.

**FIGURE 3:**
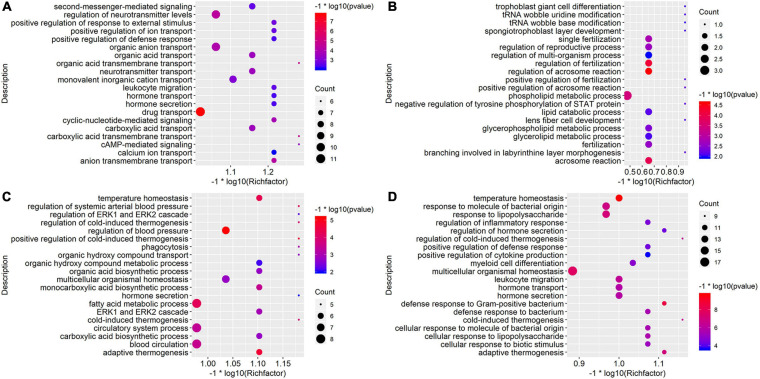
Gene Ontology (GO) enrichment analysis of down-regulated DEGs in comparison groups. The top 20 significant Biological Processes terms of down-regulated DEGs in D10NTC vs. D10TOA group **(A)**, top 20 significant Biological Processes terms of down-regulated DEGs in D10NTC vs. D20TOA group **(B)**, top 20 significant Biological Processes terms of down-regulated DEGs in D30NTC vs. D30TOA group **(C)**, and top 20 significant Biological Processes terms of down-regulated DEGs in D30NTC vs. D35TOA group **(D)**. Size and color of the bubbles are measured as count and *p-*value to represent the amount of DEGs enriched in these terms and the enrichment significance.

To evaluate the global picture of the mice intestine transcriptomic response to *L.casei* treatment and gene functions related to antimicrobial peptides, GO Biological Processes term was performed by using DEGs in comparison groups. We took the top 20 significant GO terms of every comparison group (shown in Figures 2, 3). On Biological Process level, up-regulated DEGs in D10NTC vs. D10TOA group were most enriched in interspecies interaction between organisms, myeloid cell differentiation, T cell activation, and regulation of innate immune response (Figure 2A); up-regulated DEGs in D20NTC vs. D20TOA group were primarily associated with GO terms regulation of steroid metabolic process, leukocyte cell-cell adhesion, and regulation of T cell activation (Figure 2B). Up-regulated DEGs in D30NTC vs. D30TOA group were most enriched in adaptive immune response, T cell activation, humoral immune response, and positive regulation of cytokine production (Figure 2C); up-regulated DEGs in D30NTC vs. D35TOA group were most enriched in sulfur compound metabolic process, adaptive immune response, and lymphocyte differentiation (Figure 2D).

In a similar way, down-regulated DEGs in D10NTC vs. D20TOA group were most enriched in drug transport, regulation of neurotransmitter levels, and organic anion transport (Figure 3A); down-regulated DEGs in D20NTC vs. D20TOA group were primarily associated with GO terms regulation of phospholipid metabolic process, regulation of acrosome reaction, and regulation of fertilization (Figure 3B). Down-regulated DEGs in D30NTC vs. D30TOA group were primarily associated with GO terms regulation of fatty acid metabolic process, blood circulation, and circulatory system process (Figure 3C); down-regulated DEGs in D30NTC vs. D35TOA group were most enriched in multicellular organismal homeostasis and response to lipopolysaccharide (Figure 3D).

Also we found out the antimicrobial-peptides-related GO terms were strongly associated with up-regulated DEGs. There were three genes, namely *Defa39*, *Defa17*, and *Reg3a*, in D10NTC vs. D10TOA group; six genes- *Cxcl13*, *Reg3g*, *Defa35*, *Reg3b*, *Defa27* and *Defa38*-were identified in D30NTC vs. D30TOA group.

### Time Series Analysis of Antimicrobial Peptide Expression

**FIGURE 4:**
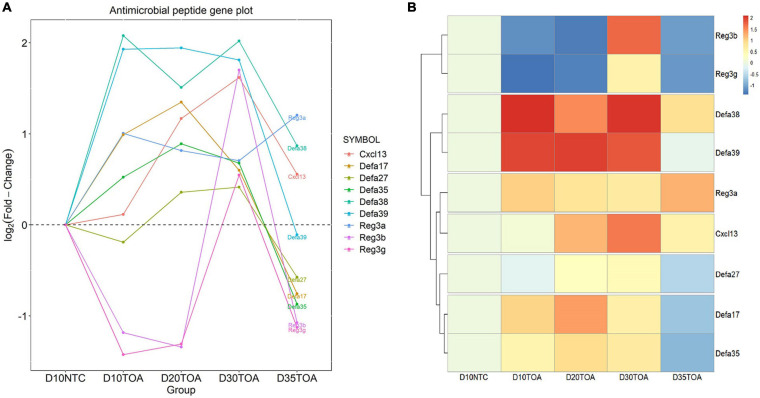
Time Series Analysis of antimicrobial peptide expression. Time trend analysis of antimicrobial peptide expression was followed by the STEM software. Time trend curve was visualized by R software with ggplot2 package **(A)**, and cluster analysis was visualized by R software with pheatmap package **(B)**.

In order to understand the expression of antimicrobial peptides at each time point, we analyzed the time trend of antimicrobial peptide expression. We found that the antimicrobial peptide *Reg3a* gene was up-regulated at each time point with a flat expression trend, especially at 5 days of non-continuous oral feeding (Figure 4A). We also got the same conclusion from the cluster analysis (Figure 4B). So we chose *in vitro* prokaryotic expression of this gene and probed its antibacterial and proliferation promoting functions.

### Validation of RNA-Seq by qRT-PCR

**FIGURE 5:**
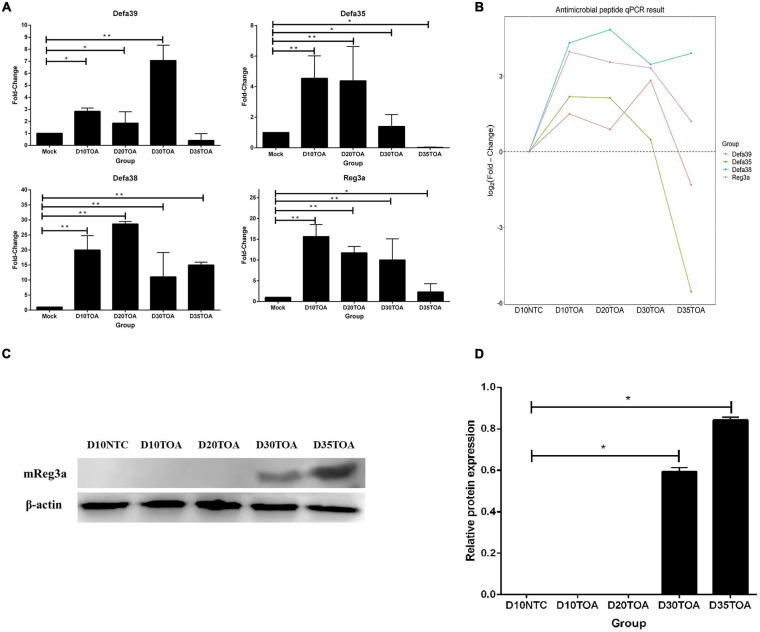
Relative quantification of DEGs for verification by qRT-PCR. The relative quantification of Defa35, Defa38, Defa39, and *Reg3a* by qRT-PCR was calculated by 2^– △△*Ct*^ method **(A)**. The relative quantification data was used to show the time trend analysis **(B)**. Western blot data **(C)** were performed with bar plot by gray analysis **(D)**. Error bars indicate standard deviations. *p < 0.05, **p < 0.01, and ***p < 0.001 using Student’s *t*-test to evaluate differences between every two groups.

To verify the results of RNA-seq identification, we performed qRT-PCR analysis of four antimicrobial peptides randomly (Figure 5A). Time trend analysis showed that the expression pattern detected by qRT-PCR was consistent with the RNA-seq results (Figure 5B). Although the fold change in the expression patterns of RNA-seq and qRT-PCR was slightly biased, probably owing to methodological differences, these results suggested that our RNA-Seq data were reliable.

In order to identify the expression of *mReg3a* protein in mice intestine, we used Western blot for mice intestine samples. Western blot analysis found out the expression of *Reg3a* protein was significantly increased in D30TOA and D35TOA (*p* < 0.05) (Figures 5C,D). D10NTC, D10TOA, and D20TOA samples have no band because of their low expression.

### Prokaryotic Expression of the Antimicrobial Peptide *Reg3a* Genes From Intestinal Tissue of Mice

**FIGURE 6:**
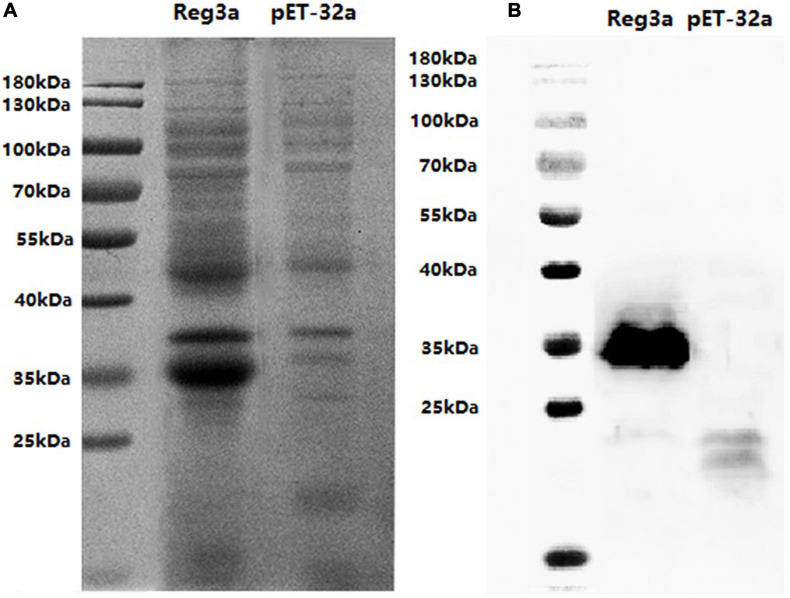
Prokaryotic Expression of the antimicrobial peptide *Reg3a* Genes. SDS-PAGE analysis for the expression of recombinant proteins in E.coli BL21 with pET-32a vector. The inducing expression of *Reg3a* was shown in the recombinant vector and not in empty vector **(A)**. The Western bolt result was as the same as the PAGE **(B)**.

The prokaryotic expression of the antimicrobial peptide *Reg3a* Genes was performed in *E. coli* strain BL21 (DE 3.0) by pET-32a vector. All of the recombinant proteins could be detected by the induction with 1 mM IPTG in 30°C, and the molecular weight of recombinant proteins was consistent with their theoretic values (35 KDa) (Figure 6A). Western bolt analysis showed that the recombinant proteins molecular weight (35 KDa) was consistent with their calculated values (Figure 6B). Since the recombinant protein was soluble, subsequent experiments were performed after protein purification. The concentration of purified protein was 4.3 mg/ml, and the purity of protein was 90% by Bio-Rad image Lab software.

### The Recombinant Antimicrobial Peptide Could Inhibit Bacteria and Virus Proliferation *in vitro*

**FIGURE 7:**
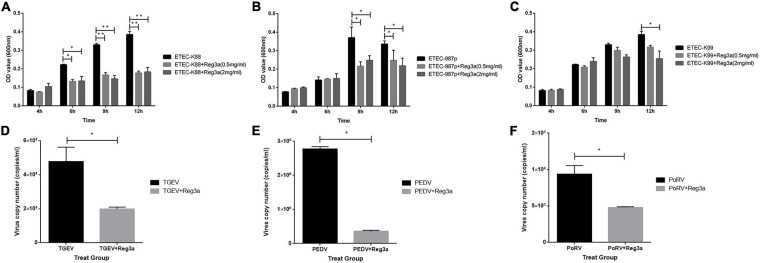
Effect of the expressed antimicrobial peptide on bacteria and virus. For understanding the effect of m*Reg3a* on bacteria and viruses, we used ETEC-K88 **(A)**, ETEC-987p **(B)**, ETEC-K99 **(C)**, TGEV **(D)**, PEDV **(E)**, and PoRV **(F)**, which are often found in infected piglets, to co-culture with *Reg3a* protein. Error bars indicated standard deviations. *p < 0.05, **p < 0.01, and ***p < 0.001 using by Student’s *t*-test was used to evaluate differences between every two groups.

In order to understand the effect of recombinant antimicrobial peptide *Reg3a* on bacteria and viruses, we used Enterotoxigenic *Escherichia coli* strain K88 (Figure 7A), 987p (Figure 7B), K99 (Figure 7C), Porcine transmissible gastroenteritis virus (TGEV), Porcine epidemic diarrhea virus (PEDV), and Porcine rotarvirus (PoRV), which can infect piglets. The antimicrobial peptide protein *Reg3a* was set at a final concentration of 0.5 and 2 mg/ml for co-culture with bacteria. We found that antimicrobial peptide *Reg3a* significantly inhibited the proliferation of ETEC-K88, ETEC-987p, and ETEC-K99 within 6, 9, and 12 h in final concentration of 2 mg/ml, respectively (*p* < 0.05). However, antimicrobial peptide *Reg3a* significantly inhibited the proliferation of ETEC-K88 and ETEC-987p within 6 and 9 h in a final concentration of 0.5 mg/ml (*p* < 0.05), and there was no significant inhibition effect on ETEC-K99 at any time points in 0.5 mg/ml *Reg3a*.

To inhibit the effect on porcine diarrhea virus, we conducted 2 mg/ml antimicrobial peptide protein for co-culture with TGEV (Figure 7D), PEDV (Figure 7E), and PoRV (Figure 7F) for 12 h. The results showed that the virus copy numbers in the co-culture group was significantly lower than that in the virus control group, suggesting that the antimicrobial peptide *Reg3a* significantly inhibited the proliferation of TGEV, PEDV, and PoRV.

### The Antibacterial Peptide *Reg3a* Promoted the Proliferation of Porcine Intestinal Epithelial Cells and Mouse Intestinal Epithelial Cells

**FIGURE 8:**
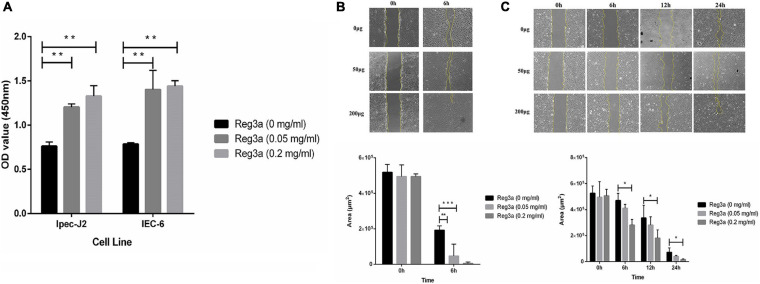
The result of CCK8 assay and healing assay. The recombinant antimicrobial peptide *Reg3a* treated the Ipec-j2 cells and IEC-6 cells within 24 h. No antimicrobial peptide was used as control **(A)**. For healing assay, the recombinant antimicrobial peptide *Reg3a* treated Ipec-j2 cells **(B)** and IEC-6 cells **(C)** in 24 h. No antimicrobial peptide was used as control. Error bars indicate standard deviations. *p < 0.05, **p < 0.01, and ***p < 0.001 were compared by Student’s *t*-test to evaluate differences between every two groups.

To understand whether the recombinant antimicrobial peptide *Reg3a* promotes the proliferation of porcine intestinal epithelial cells and mouse intestinal epithelial cells, cytotoxicity kit-8 assay (CCK8) and healing assay were performed in this experiment. The CCK8 assay indicated that the *Reg3a* treatment final concentration of 0.05 and 0.2 mg/ml significantly promoted the cells Ipec-J2 and IEC-6 proliferation after 24 h (*p* < 0.05) (Figure 8A). For healing assay, we found that the wounded area of the Ipec-j2 cells was significantly smaller than that in the control group under a final concentration of 0.05 and 0.2 mg/ml in the *Reg3a* treat group in 6 h (*p* < 0.05) (Figure 8B). However, the wounded area of IEC-6 cells in *Reg3a* treat group was significantly smaller than that in the control group only with a final concentration of 0.2 mg/ml within 6, 12, and 24 h (*p* < 0.05) (Figure 8C).

**FIGURE 9:**
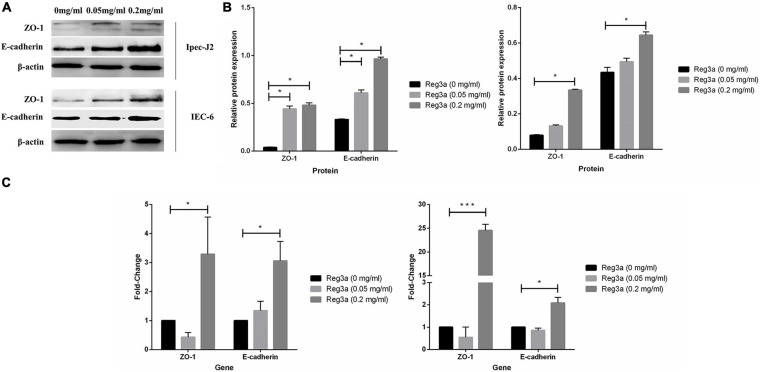
Relative quantification of tight junction protein by Western Blot and qRT-PCR. The cells Ipec-j2 and IEC-6 were treated with the recombinant antimicrobial peptide *Reg3a* within 24 h. There was no antimicrobial peptide as control. Western blot data **(A)** were performed with bar plot by gray analysis **(B)**. For qRT-PCR assay **(C)**, relative quantification of tight junction proteins were calculated by 2^– △△*Ct*^ method. Error bars indicate standard deviations. *p < 0.05, **p < 0.01, and ***p < 0.001 were compared by Student’s *t*-test to evaluate differences between every two groups.

In order to discuss whether the recombinant antimicrobial peptides *Reg3a* promotes the expression of tight junction protein or not in porcine intestinal epithelial cells and mouse intestinal epithelial cells, Western blot and qRT-PCR assay were carried out in this experiment. From the Western blot analysis, we found out the expression of both tight junction protein ZO-1 and E-cadherin was significantly increased in *Reg3a* treatment group with 0.05 and 0.2 mg/ml in Ipec-J2 cells (*p* < 0.05). Nevertheless, the tight junction protein ZO 1 and E-cadherin expression were significantly increased in *Reg3a* treatment group only with the final concentration of 0.2 mg/ml in IEC-6 cells (*p* < 0.05) (Figures 9A,B). The qRT-PCR results indicated that the two tight junction proteins ZO 1 and E-cadherin in *Reg3a* treatment group were significantly higher than that in the control group under the final concentration of 0.2 mg/ml both in Ipec-J2 and IEC-6 cells (*p* < 0.05) (Figure 9C).

## Discussion

Probiotics have been used extensively as animal feed additives. There are several benefits set for probiotics: protection and prevention of infections (Hu et al., 2017; Azad et al., 2018), prevention of cancer (So et al., 2017), and promotion of food absorption and repair of intestinal epithelial barrier (Abraham and Quigley, 2017; Kim et al., 2018). Our studies on *L.casei*, one of the probiotics, as a live vehicle for the delivery of heterologous antigens to the mucosa have been conducted for over 20 years. But we are still unclear on what antimicrobial peptides are produced by *L.casei* stimulation and what function they have on the diarrheal bacteria and viruses and damaged intestine epithelial cells. The bioinformatics analysis on RNA-seq revealed there were nine genes relative to the high expression of antimicrobial peptides after intragastric administration of *L.casei* to mice. However, it is only *Reg3a* that is persistently highly expressed at each time point. So we chose this antimicrobial peptide to express *in vitro* in order to explore its activity on pathogenic microorganisms and wound healing ability. We expected that this antimicrobial peptide could protect baby animals against the gut pathogens transmitted via mucosa.

In our study, GO analysis in biological processes showed that DEGs were mostly annotated for T cell activation, humoral immune response, and regulation of innate immune response. This result indicated that *L. casei* treatment may activate the body’s immune response, especially humoral immune response. Similar results were reported in some studies demonstrating that *L.casei* could activate the body’s humoral immunity and enhance the body’s resistance to pathogenic bacteria (Ogawa et al., 2006; Núñez et al., 2014; Asghar et al., 2016; Ren et al., 2020). GO analysis also indicated that more antimicrobial-peptides-related GO terms were associated with up-regulated DEGs and mostly belong to the defensin family. It means that *L.casei* treatment may increase the antimicrobial-peptides gene expression. Similar results were shown in some studies and indicated that *Lactobacillus* strain treatment increased the mRNA abundance of porcine β-defensin 2 (pBD2) and pBD3 in the piglet’s jejunum and ileum (Zhang et al., 2011; Wang Q. et al., 2019). In a time series analysis of antimicrobial peptide expression, we found that the most antimicrobial peptide gene in GO terms which enriched significantly by DEGs was up-regulated within days 10 with *L*.*casei* treatment. It does also prove that *L.casei* treatment may increase the antimicrobial-peptides gene expression. However, some antimicrobial-peptides genes had a decreased expression 5 days post-treatment. One possible reason is that after the discontinuation of *L. casei* treatment, the intestinal flora gradually returned to its pre-treatment status. Another possible reason is the stimulation of *L.casei* to the enterocytes was decreased or disappeared. But there are a very limited number of studies that have analyzed this status and further studies are still needed to prove this.

In order to understand the antibacterial activity of antibacterial peptide, we expressed the antibacterial peptide m*Reg3a in vitro*. The co-culture experiment result indicated that recombinant antibacterial peptide m*Reg3a* can inhibit bacteria and virus proliferation *in vitro*. More studies have shown that the Reg family, which belonged C-type lectin secreted by Paneth cells, has powerful activity against bacteria and viruses of the gut (Cash et al., 2006; Mukherjee et al., 2014). Also, as host defense, antibacterial peptide m*Reg3a* can kill Gram-positive bacteria and play a vital role in antimicrobial protection of the mammalian gut (Mukherjee et al., 2009; Lehotzky et al., 2010; Natividad et al., 2013). Nevertheless, there were fewer studies about m*Reg3a* against other pathogens, and we need more experiments to test its anti-pathogen function.

The CCK-8 assay and healing assay result indicated that *Reg3a* can promote the proliferation of porcine intestinal epithelial cells and mouse intestinal epithelial cells. Similar results were reported in some studies demonstrating that *Reg3a* could regulate keratinocyte proliferation and differentiation after skin injury (Lai et al., 2012). Our results also showed that *Reg3a* can promote tight junction protein expression in intestinal epithelial cells. However, there were fewer reports that *Reg3a* can promote the proliferation of intestinal epithelial cells. Mostly studies indicated that *Reg3a* can alter the intestinal microbiota and control inflammation (Darnaud et al., 2018; Zhao et al., 2018).

Thus, oral administration of *L.casei* can induce intestinal cells to express antimicrobial peptides, which can promote the proliferation of intestinal epithelial cells. To sum up, this study revealed that the antimicrobial peptide which *Lactobacillus casei* induced was worthwhile to promote intestinal cell proliferation and repair in piglets. However, it is still necessary to conduct further experimental studies to uncover antimicrobial peptide impact intestinal *in vivo*, and on the molecular mechanism and cellular pathway of repairing host intestinal mucosal barrier. This proliferative and reparative effect is a strategy to prevent intestinal mucosal damage due to diarrhea in piglets.

## Data Availability Statement

The original contributions presented in the study are publicly available. This data can be found here: NCBI repository, accession number: PRJNA719601.

## Ethics Statement

The animal study was reviewed and approved by the Heilongjiang Bayi Agricultural University Institutional Animal Care and Use Committee.

## Author Contributions

YB and LY designed the experiments. YB conducted the experiments. YB and YH analyzed the experimental results. YB, YH, and YL analyzed the RNA sequencing data. YB and YL used Graphpad prism 8.0 software to plot the experimental results. YB and YH wrote the manuscript. LY, BZ, CX, and XH reviewed and modified the manuscript. All authors contributed to the article and approved the submitted version.

## Conflict of Interest

The authors declare that the research was conducted in the absence of any commercial or financial relationships that could be construed as a potential conflict of interest.

## Footnotes

1 http://www.bioconductor.org

2 http://www.bioconductor.org/packages/release/bioc/html/DESeq2.html
